# Deep neural network recognition of shallow water corals in the Gulf of Eilat (Aqaba)

**DOI:** 10.1038/s41598-020-69201-w

**Published:** 2020-07-31

**Authors:** Alina Raphael, Zvy Dubinsky, David Iluz, Jennifer I. C. Benichou, Nathan S. Netanyahu

**Affiliations:** 1 Faculty of Life Sciences, The Mina and Everard Goodman, Bar-Ilan University, 5290002 Ramat-Gan, Israel; 2 Department of Computer Science, Bar-Ilan University, 5290002 Ramat-Gan, Israel; 3 Department of Environmental Sciences and Agriculture, Beit Berl College, 4490500 Beit Berl, Israel

**Keywords:** Ecology, Ecosystem ecology, Ecology, Ecosystem ecology

## Abstract

We describe the application of the computerized deep learning methodology to the recognition of corals in a shallow reef in the Gulf of Eilat, Red Sea. This project is aimed at applying deep neural network analysis, based on thousands of underwater images, to the automatic recognition of some common species among the 100 species reported to be found in the Eilat coral reefs. This is a challenging task, since even in the same colony, corals exhibit significant within-species morphological variability, in terms of age, depth, current, light, geographic location, and inter-specific competition. Since deep learning procedures are based on photographic images, the task is further challenged by image quality, distance from the object, angle of view, and light conditions. We produced a large dataset of over 5,000 coral images that were classified into 11 species in the present automated deep learning classification scheme. We demonstrate the efficiency and reliability of the method, as compared to painstaking manual classification. Specifically, we demonstrated that this method is readily adaptable to include additional species, thereby providing an excellent tool for future studies in the region, that would allow for real time monitoring the detrimental effects of global climate change and anthropogenic impacts on the coral reefs of the Gulf of Eilat and elsewhere, and that would help assess the success of various bioremediation efforts.

## Introduction

One of the major challenges in the field of contemporary ecology is the documentation of ecosystem change over time. Among coastal marine biota, coral reefs are home to a unique hotspot of biodiversity. In the last decades, coral reefs are undergoing a severe decline worldwide^1–3^ due to a combination of ocean acidification^4,5^, and seawater warming^6,7^, their adverse impacts intensified by anthropogenic eutrophication and pollution^8^. These bring about both the decline in live reef cover and a decrease in coral species diversity^1^. Hence it is of paramount importance to monitor and document the rates of reef decline and identify the relative importance of stressors in each reef. An additional benefit of automated analysis of reef images is its potential as a tool to evaluate the long-term success of bioremediation projects of damaged coral reefs^9^ and reef protection measures.

The use of artificial intelligence (AI) to solve the time-consuming, tedious manual classification of coral species and determination of their abundance in real-time, is a Herculean task by itself due to the immense numbers of necessary images and their examination. Automated Deep learning (DL), a branch of AI, has the potential of solving this problem efficiently, and by far exceeds in terms of reliability and accuracy human reef documentation and monitoring. Like the human brain, the more data the computer learns under the DL mechanism, the better it performs at distinguishing among classes of coral species in the present application.

The highly-diverse coral reefs of the Gulf of Eilat (Aqaba) are of special scientific interest not only for being the most Northern reefs, but for benefiting the economy of neighboring communities in both Israel and Jordan. Although highly-diverse^10^, these reefs have suffered from a sequence of disasters, including a rare low tide in 1970^11^, the recovery from which was impeded by repeated oil pollution following the closure of the Suez Canal between 1967 and 1975^12^. The subsequent recovery of the gulf’s reefs was slowed down again by the episode of the cooling of the Gulf’s waters due to the eruption of Mount Pinatubo in 1991. That event caused erosion of the Gulf's thermocline and led to deep mixing of its waters, enriching surface waters with nutrients. The resulting proliferation and subsequent decomposition of seaweeds smothered some 25% of the juvenile corals^13^. The anthropogenic eutrophication of the Gulf due to the increase in fish farming until the farm closing^14^, also reduced the transparency of its waters by increasing the concentration of phytoplankton^15^. These events, as well as the forthcoming Red-Dead Canal, call for frequent, detailed monitoring of any changes in the situation of the coral reefs of the Gulf, as are evident in the reduction of live coral cover and species biodiversity.

Since its early development, DL has been used in human facial discrimination^16^, handwriting recognition^17^, and forensic applications, such as fingerprint identification^18^ and voice analysis^19^.

DL has also been applied to various coral reef studies, in which it was used to discriminate among benthos types: sand, urchins, and three types of branched corals: brain coral, massive favids, and dead coral^20^, as well as to distinguish between healthy and bleached corals (see, e.g.,^21,22^). Shihavuddin et al.^20^ demonstrated the capability of DL to identify five coral genera from large assemblages of underwater images. In a recent study identification among branched and brain corals, was reported^23^.

In their research, Gómez Ríos et al.^23^ also distinguished among favids, brain coral, and three branched coral types: I, II, and III (an urchin, dead corals, and pavements based on an image mosaic).

Among DL studies at species level discrimination the following datasets are noteworthy:

The first dataset is the Pacific Labelled Corals (PLC) dataset, which contains 5,090 images from four locations: 1. Mo'orea (French Polynesia), 2. Northern Line Islands, 3. Nanwan Bay (Taiwan), and 4. Heron Reef (Australia). The PLC dataset contains: 251,988 annotations from these four locations made by a coral reef expert using a random point tool. In addition, six experts cross-annotated 200 images from each location^21^.

The second dataset is the Mo'orea Labeled Corals (MLC) dataset that includes five coral classes: *Acropora*, *Pavona*, *Montipora*, *Pocillopora*, and *Porites*, and four non-coral classes: crustose coralline algae, turf algae, macroalgae and sand. The MLC dataset contains over 400,000 human expert annotations of 2055 Mo'orea island survey images (https://vision.ucsd.edu/datasetsAll).

To apply DL to a coral reef dataset, there is no need to sample small fragments of corals for the subsequent tedious identification in the laboratory. DL enables direct classification of a large amount of photographs, in minimum time.

The novelty of the present study is the application of a fully-automated DL-based methodology to an imaging dataset, for the classification of 11 common coral types from the set of species reported in the Gulf of Eilat^11^. We applied DL to over 5,000 underwater images taken specifically by us from a shallow reef in the Gulf of Eilat, with the aim of documenting the distribution of the test types in the sample reef.

We demonstrated the power of DL, using shallow coral reefs in the Gulf of Eilat for comparison with its efficiency in other reef sites. That site allowed us to compare our DL data with the detailed manual coral surveys previously conducted on these reefs.

### Corals and reefs

The nature and global importance of corals and the rapid destructive impact of Global Climate Change call for extensive and fast indexing and monitoring. Coral reefs cover less than 1% of the total area of the oceans and seas, yet they are the main repository of oceanic biodiversity (25% of all marine species)^24^. Extant hermatypic (reef-building) coral species are estimated at 3,235^24^, of which 100 were recorded in Eilat, Gulf of Aqaba, Northern Red Sea^10^. Hexacorals, based on six fold symmetry, or scleractinian corals are the most important hermatypic organisms^25^.

The decline of reefs leads to the collapse of their entire complex ecosystem depending on the calcium carbonate skeletons of the corals intricate reef structures, for food and shelter. Hermatypic corals are home to symbiotic algae living within their cells in specialized organelles, the symbiosomes. Called zooxanthellae by their first reporter, Brandt^26^, these greenish microalgae limited to sunlit shallow waters (~ 0–120 m), provide the corals with energy through their photosynthesis^27,28^, which also stimulates calcification^29^.

In most corals, the tentacles are retracted by day and spread out at night^30^ to catch plankton and other small organisms, while avoiding diurnal coral-feeding predators. This behaviour also optimizes the supply of oxygen for nocturnal respiration.

Unlike in shallow water, corals satisfy their energy needs in the deep water and dim light by zooplankton consumption, as an energy supplement to the algal light-limited photosynthetic products (see review by Dubinsky and Iluz^28^).

The photosynthetic activity of the zooxanthellae, raises the internal pH of the coral facilitating the skeletal calcification by "light enhanced calcification"^31,32^, a paradigm recently challenged by Cohen et al.^33^. Conversely, ocean acidification makes coral calcification more difficult.

### The future of coral reefs

Coral reefs are exposed to many dangers because of global climate-change effects^34,35^, blast and cyanide fishing^36^, coral collection by the marine coral aquarium trade^37^, sunscreen use^38^, and light pollution interference with lunar cycle reproduction timing^39^. SCUBA diving pressure^40^. Anthropogenic eutrophication, acts synergistically with all the above listed detrimental factors, stimulating fast seaweed growth, that easily outcompete the slowly growing corals. The ensuing algal blooms, smother the coral colonies and prevent the settlement of juveniles^41^. Kaneohe Bay, a coral reef ecosystem at Oahu, Hawaii, illustrates the sensitivity of coral reefs to nutrient enrichment resulting from treated sewage disposal, leading to the reversible proliferation of seaweeds^42^. Fish cage farming released nutrients that affected the coral reefs in Eilat by causing deterioration in water quality due to eutrophication and by promoting seaweed growth and phytoplankton proliferation reducing the Gulf’s water transparency, thus reducing light necessary for symbiont photosynthesis, interfering with reproduction, increasing bio-erosion and epizootic infestation^14^.

Coral species differ in their tolerance to climate change and coral bleaching^43^. Corals experience bleaching as water temperature increases and causes loss of the zooxanthellae, and subsequently of live coral tissue, resulting in wide spread coral mortality followed by reef destruction. Unless the algal population recovers within weeks, the bleaching results in widespread reef mortality^44^. The ongoing increase in atmospheric carbon dioxide since the industrial revolution leads to ocean acidification or lowering of ocean pH, and affects corals negatively by shifting the balance from skeletal aragonite deposition toward its dissolution^4^. In addition, light pollution by artificial light, even at the weakest intensities^45,46^, can cause the disruption of coral reproduction that is controlled by lunar periodicity^47,48^. The planned Red Sea–Dead Sea Conveyance^49^ will cause a change in the regime of the Gulf currents^50^. Such a change could reduce the supply of larvae of corals and other reef organisms, and have a far-reaching deleterious impact on reef systems.

The real-time characteristics of DL tools are crucial for the rapid detection of reef damage allowing implementation of bioremediation measures. The DL characteristics are valuable tools assuring the health and long-term survival of the coral reefs in the Gulf of Eilat and worldwide.

### Deep learning

The efficiency of the methodology of DL-based classification of coral species consists of efficient algorithms that reveal and extract common-patterns and features from large image datasets. Two popular schemes applied to coral reef data are the convolutional neural network (CNN)^21^ and deep belief net (DBN)^21^. A generic structure of CNN is a multi-layer, feed-forward, supervised neural network that recognizes objects from spatial-based images with little or no pre-processing. It consists of: (1) feature extraction (convolution layer); (2) distortion invariance (sub-sampling layer); and (3) classification (output layer). A DBN, consists of probabilistic models composed of multiple layers of random variables^51^.

Any coral-reef classification should consist of five main steps:

1. Taking sufficient high quality underwater images.
2. Detecting the chosen coral and cropping its image.
3. Downscaling the cropped images to 200 × 200 pixels.
4. Preprocessing the images to compensate for different imperfections (e.g., blurring, colour change, sunlight wave patterns, sky colour, nekton scattering effects etc.).
5. Labelling each one of the coral species is labelled.

In case of an automatic model, Steps 3 and 4 may not be required.

Traditional machine learning methods need extensive domain expertise, human intervention, and are only capable of what they were originally designed for.

Additional works on growth modelling and quantification of morphological variation in coral types are due to, Kruszyński et al.^52^ and Chindapol et al.^53^. The former focused on the analysis of three-dimensional (3D) coral images scanned by X-ray tomography, and the latter, modelled the effects of flow on colony growth and shape, using analyzed advection–diffusion equations.

The increased interest in DL has also been recently reflected in the analysis of previously published coral datasets. Specifically, recent work^22^ has demonstrated the efficiency of neural networks and DL in distinguishing among various marine benthos components such as bare ground, seagrass meadows, algal cover, sponges, and identified some coral species. Additional recent work has shown the capability of neural networks and DL to distinguish among coral species and live corals from bleached colonies (see, e.g.,^21,22^).

Mahmood et al.^22^ combined CNN representations with manually obtained colony parameters. Their algorithms, based on image information, extract CNN images obtained from the deep VGGnet network with a 2-layer multilayer perceptron (MLP) classifier (trained on the MLC dataset). They achieved 77.9% accuracy.

Mahmood et al.^54^, reviewed the power of DL for machine monitoring of coral reefs.

Mahmood et al. (2016a)^55^ reported a decrease trend in coral density and species numbers in the reefs of Abrolhos Islands. Their analysis was based on CNN images obtained from VGGnet. They proved the reliability of their classifier on unlabelled coral image mosaics.

Mahmood et al. (2018)^56^ used CNN-based features and ResFeats to annotate corals and demonstrated the temporal changes in their association. They applied generic features from VGGnet and ResNet to classify corals and non-corals. They analysed unlabeled coral mosaics of three Abrolhos Island sites generating maps for the aforementioned mosaics.

Mahmood et al. (2020)^57^ applied computerized DL characterization of annotated kelp species. They presented an automatic hierarchical classification method to classify kelps in collected images. Their study summarises the considerable advantages of using deep residual networks (ResNets) over traditional, manual classifications of the same reefs. They showed that the sibling hierarchical training approach outperforms the traditional parallel multi-class classifications by a significant margin (90.0% vs. 57.6% and 77.2% vs. 59.0%) on Benthoz15 and Rottnest datasets, respectively. They used an application to study the changes in kelp cover over time for annually repeated AUV surveys.

Mahmood et al. (2020)^58^ evaluated how well features extracted from deep neural networks transfer to underwater image classification. They investigated the effectiveness of transfer learning of the ResFeats. They proposed applying new image features (called ResFeats) extracted from the different convolutional layers of a deep residual network pre-trained on ImageNet to the MLC, Benthoz15, EILAT and RSMAS datasets.

Gómez-Ríos et al.^23^ included more corals than previous studies by applying three CNNs: Inception v3^59^, ResNet^60^, and DenseNet^61^ (see Supplementary Table 1).

Two datasets were analysed: Both the EILAT and RSMAS were analysed. These datasets comprise patches of coral images discriminating branched and massive colonies. The EILAT dataset contains 1123 images of eight classes (sand, urchin, branched type I, II, and III corals, brain coral, favid coral, and dead coral) and the RSMAS dataset contains 776 images of 14 classes, including 9 classes of the following scleractinian coral species: *Acropora cervicornis*, *Acropora palmata*, *Diploria strigosa*, *Montastraea cavernosa*, *Meandrina meandrites*, *Montipora* spp., *Siderastrea sidereal*, *Colpophyllia natans* (a boulder brain coral), and the colonial fire coral *Millepora alcicornis* (a species of hydrozoa with a calcareous skeleton). The other five classes included in the RAMAS dataset are non-coral species: Diadema antillarum is a sea urchin, Gorgonians are a genus of soft corals in the family Gorgoniidae, Palythoas palythoa is a genus of anthozoans in the order Zoantharia, and sponge fungus and tunicates are marine invertebrates of the subphylum Tunicata.

CoralNet (https://coralnet.ucsd/edu), conceived by Beijbom et al.^9,62^, uses deep neural networks for fully- or semi-automated image annotation. It also serves as a convenient, user-friendly collaboration platform. In early 2019, Williams et al.^63^ in a large study showed that the automated annotations for CoralNet Beta, produced benthic cover estimates comparable to controls gathered by human annotation.

Hoegh-Guldberg states that “CoralNet will allow the world’s scientists to quickly assess the health of endangered coral reefs at scales never dreamed of before”, in (https://blogs.nvidia.com/blog/2016/06/22/deep-learning-save-coral-reefs/).

The BenthoBox image labelling system for ecologists allows storing images of the dataset.

The software uses learning algorithms to recognise ‘tagged’ seabed features such as sand, algae, sponges and corals.

### History of coral classification in the Gulf of Eilat

Traditional methods have been used in Gulf of Eilat research studies for coral classification since the pioneering work by Loya and Slobodkin^10^. Some 100 coral species were listed in their study.

Whenever confronted with doubt concerning the species of a certain coral underlying a transect, a small piece was sampled and manually identified by a taxonomist^11^, a tedious and destructive practice based on limited sample size.

These surveys were based on colour photographs taken by a camera with a flash attachment. Close-ups were taken by a Rolleiflex camera. A measuring tape was spread over the reef, and the divers recorded the projected length of all the organisms and substrates underneath the line transect to a resolution of 1 cm. Photographs were taken at 1 m intervals along the transect. This study was based on permanent transects photographed over a period of 20 months that yielded about 3,000 photographs of corals belonging to Loya's^11^ list of approximately 100 species. However, the author noted that many cryptic species do not show up in the photographs.

Similar additional surveys were conducted following various disturbances that affected the coral reefs of the Gulf: the 1970 low tide^64^, the repeated oil spills^65^, the Pinatubo eruption of 1991^66^, and the fish farming episode of 1995–2008^14^.

Diver-based methods for classifying corals are almost impossible underwater, and require time-consuming expertise. Furthermore, coral pigmentation and morphology are plastic changes in response to environmental forcing functions such as light and current, eliciting wide phenotypic variability^28,67^. Ever since the National Monitoring Program (https://www.iuieilat.ac.il/Research/NMPmeteodata.aspx) of the Eilat reefs was initiated (2003), annual surveys by divers have been conducted.

The images are taken at a fixed area at six reef sites, namely the North Beach, the Dekel Beach, the Eilat Ashkelon Pipeline Co. Ltd. (EAPC), the coral reserve, the Interuniversity Institute for Marine Sciences in Eilat (IUI) marine laboratory, and Taba. Each site has fixed camera brackets for five cameras, and each of these takes four images. In this way, 20 pictures are taken at each site, and 120 pictures are taken for quantitative analysis of the changes at the various sites. Monitoring is done once a year in early summer. Corals were identified as far as possible at the species level, and were also classified according to functional groups. The results are presented graphically following statistical processing. Due the disintegration of the rock to which the cameras were attached, some new sites had to be added^68^.

Automated DL seeks to avoid these difficulties, profiting from the latest advances in computerized handling of large quantities of visual images^9^. Indeed, these novel developments have been increasingly applied to the survey and analysis of coral reefs in the studies listed in Supplementary Table 2. Since all previous surveys, as well as those of the current monitoring program of the Gulf of Eilat reefs, were based on the manual and visual analysis of large numbers of photographs, we present here a first example at using automated machine-based analysis for the red sea coral reef.

## Methods

### Work process

The photos and underwater videos of transects were acquired at the coral reef reserve in the Gulf of Eilat (29°30′ N, 34°55′ E).

The methods used in the current study are:

- Natural sampling units by photographing the coral reef during daytime.
- Line transects for estimating the cover percentage at the four test sites in the Gulf of Eilat.
- Deep convolutional neural networks as an efficient classification for coral species using a supervised DL method called convolutional neural networks (CNNs).
- The Cochran–Mantel–Haenszel test was performed to compare the presence and proportions of coral species abundance, as measured by different methods across multiple sites. Post hoc analysis was performed with pairwise Fisher test with false discovery rate (FDR), which is the expected proportion of type I errors.

Species coverage percentage was estimated using a one-way ANOVA, followed by Tukey post hoc analysis.

### Study sites

**Figure 1:**
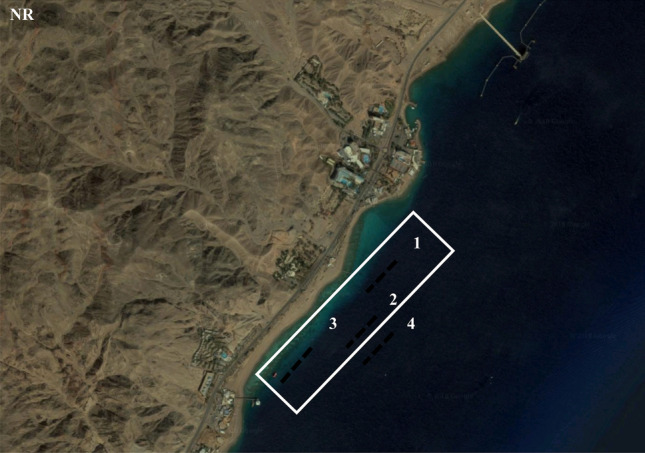
Location of study areas at the nature reserve (NR). Figure was generated from Google Maps version number: 10.26.2, URL: https://goo.gl/maps/arRzA1ZmbZvjwVJp7. Map data ©2019 Mapa GISrael, ORION-ME Imagery ©2019 , CNES/Airbus, Landsat/Copernicus, Maxar Technologies, U.S. Geological Survey.

Field work at four sites (Fig. 1) was conducted from June 2017 till June 2018 in Eilat Coral Beach Nature Reserve. This site has a well-developed reef near the shore, as well as massive stony corals throughout the entire depth gradient down to 50 m. This is the most developed, complex, and diverse coral reef in the Gulf of Eilat.

Two field data acquisition methods were used in this research:

- “**Natural sampling units**” by photographing the coral reefs.
- **Line transects** for estimating the cover percentage at the four sites in the Gulf of Eilat.

The study sites were chosen on the basis of their accessibility and central location within the Eilat Coral Reserve. Furthermore, they are highly diverse, offering the opportunity to choose the most common species. The chosen sites allow studying the variability over space (between sites), and finally, examining the possible effects of human-mediated disturbances by comparing quantity and cover percentage at the most disturbed site of the three with reference sites at different depths.

### Photography

More than a thousand still coral images were taken, and hours of underwater videos were recorded. The first step began by the underwater photographing of 400 still images, each covering about 1 m^2^ of the reef area. Subsequently, squares of 200 × 200 pixels containing any of the 4 coral genera chosen for the initial stage of the study (*Acropora*, *Favia*, *Stylophora*, *and Platygyra*) were identified visually on the computer screen, labelled, and cut out of the original images.

### Equipment

Photographing corals at the surveyed sites was done along line transects using an underwater Hero6 Black camera that offers video shooting at maximum resolution of 4K at 60 frames per second, and also supports 1080p FHD 1080P video playback, or 2.7K at 120 frames per second. The camera has video stabilization capabilities, as well as the ability to download images from the camera to a computer or smartphone through a 5 GHz WiFi connection. It also has a GPS component, accelerometer, and gyroscope.

### Transects

Each site and depth were marked by four line transects. At each site or depth, a 40 m line was laid parallel to the coastline from a randomly chosen point, and four sections were marked along the line at 10 m intervals, i.e., each section was 2.5 m. The four transects corresponded with 5, 10, 15, and 20 m depth. Every line transect produced hundreds of still photos and thousands of video frames.

### Areal coverage

In the present study, the coverage percentage of the corals serves as an indicator of the coral reef’s health. Throughout the study, the relative coverage of eleven common species was recorded.

**Figure 2:**
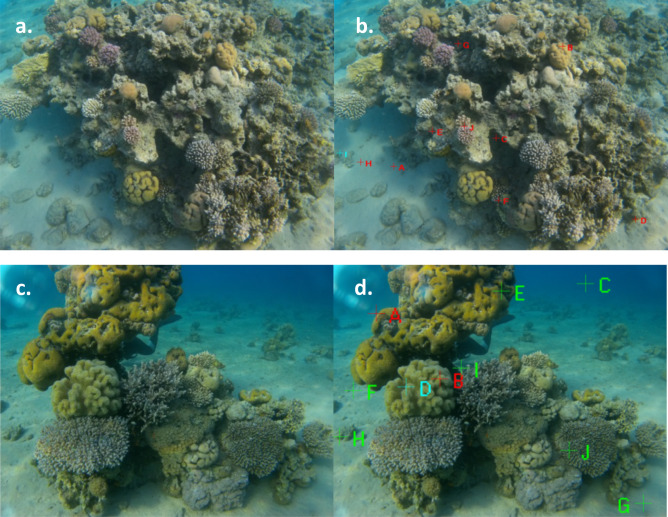
(**a**) Coral species in the Gulf of Eilat; (**b**) Coral Point Count software for the annotation process; (**c**) photograph of an additional spot of the coral reef; and (**d**) annotation process.

The results are divided into two sections:

1. Coral species quantities and coverage percentage at each site (sites 1–4) by two methods (point estimated, Fiji ImageJ) (see Fig. 2).
2. DL coral classification data.

### Sampling frequency

Field work was conducted from June 2017 till June 2018.

Transects were photographed every month until June 2018.

The images were analysed using DL on a computer with a Tesla K80 GPU accelerator.

### Cover percentage

Live coral cover was checked at the four test sites using the method of line transects. In each photo, the exact counts and cover percentage of the eleven coral species were noted for the number of these coral species per transect and for the percentage of coral coverage of each species. Examination of the photos focused on healthy corals. Count-based measurements followed the “center rule” scheme, as suggested by Zvuloni et al.^69^.

In this work, only corals with centers lying within the sampling unit are counted, and all other corals are ignored. The advantage of this technique is that the size of a coral does not play any role in the sampling probability, making this method nonbiased in contrast to other, biased, methods and corrections (reviewed by Zvuloni et al.^69^).

Cover percentage was calculated using Microsoft Excel software and CPCe 4.1 software in order to facilitate the logistics of the manual annotation process. Coral counting was done by Fiji ImageJ software. Statistical analysis was done using R statistics software.

### Sample images

#### Video

##### Sample video

Video sequences were filmed from June 2017 till June 2018 at the sites at different depths (5 m, 10 m, 15 m, and 20 m). In order to get separate photos from the videos, QuickTime software was used.

#### Use of video images

**Figure 3:**
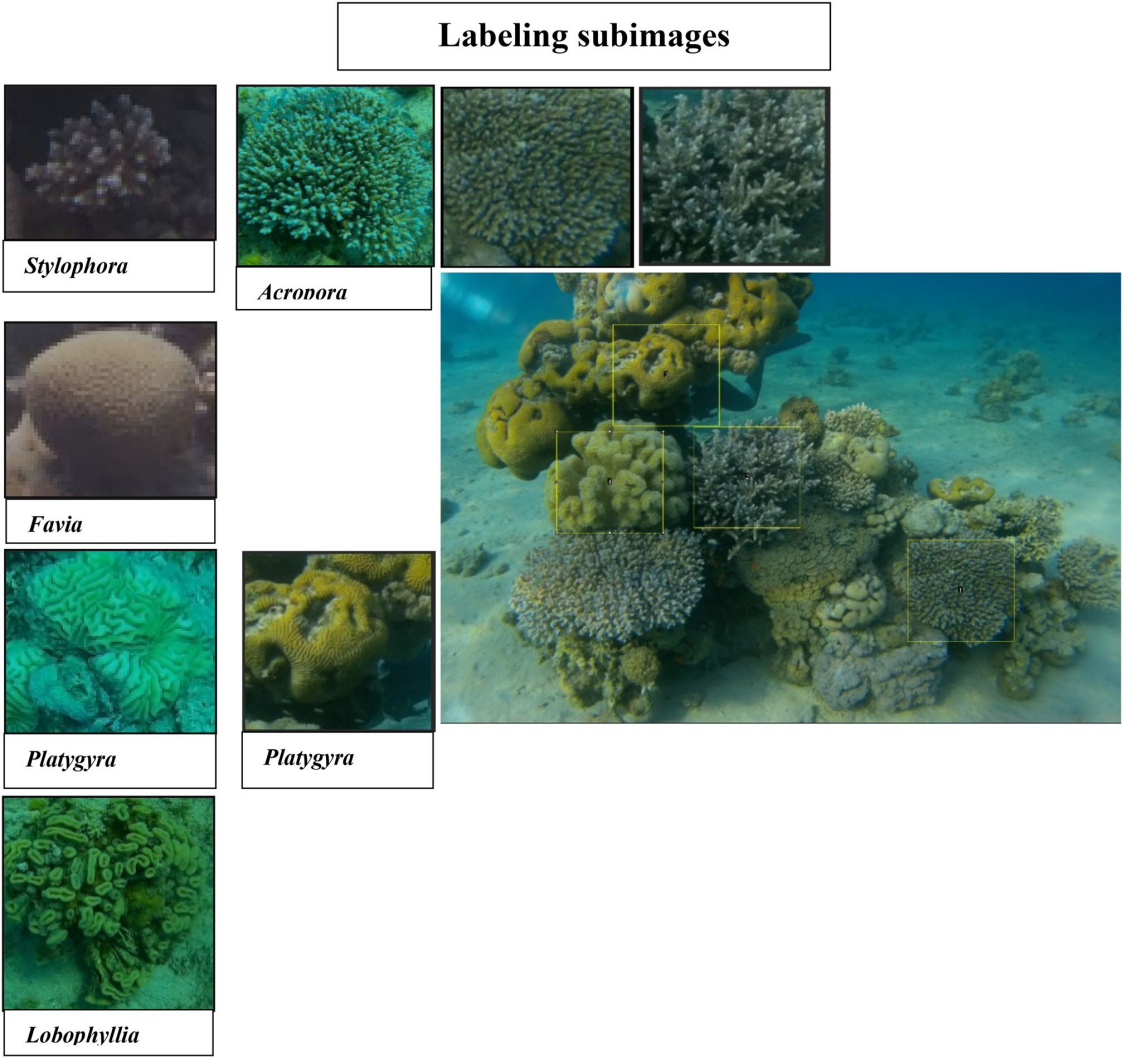
Samples of four of the coral species in the study.

Underwater videos of the coral reef species from the Gulf of Eilat were filmed in order to produce a large dataset of images. Image blocks of 200 × 200 pixel-sized image frames were manually cut to comprise the chosen training dataset of 3,850 sub images of some coral species, as shown in Fig. 3.

### Common coral species

#### Image preprocessing

**Figure 4:**
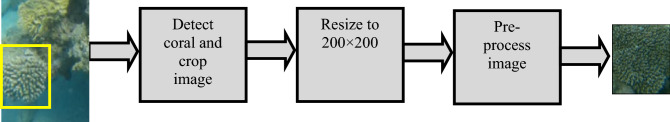
Preprocessing of an image.

**Figure 5:**
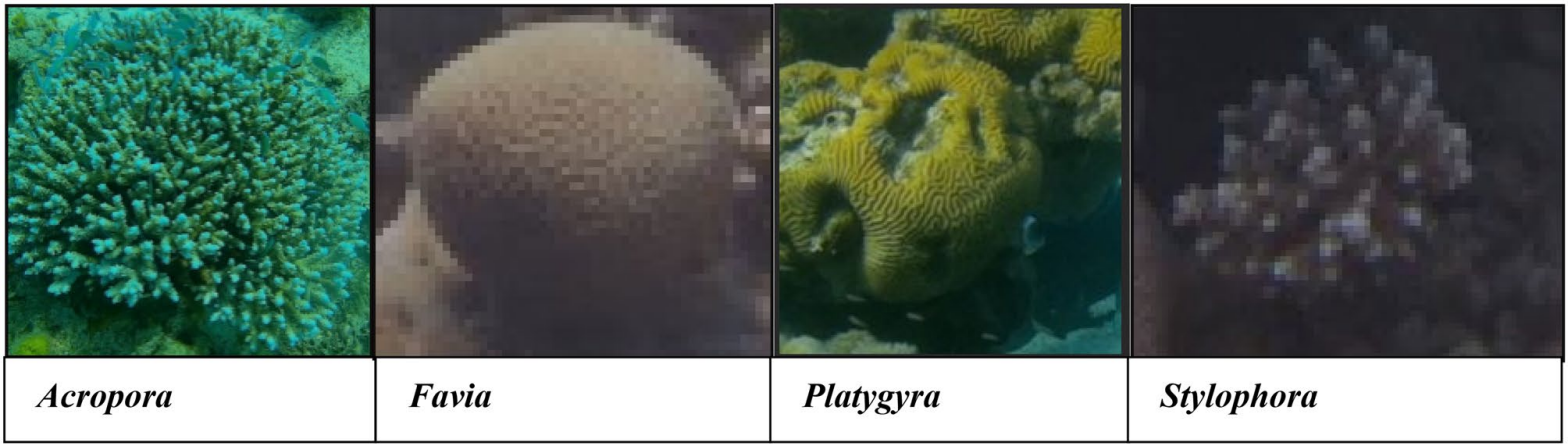
Examples of preprocessed coral images and their labels (from left to right): *Acropora*, *Favia*, *Platygyra*, *and Stylophora*.

For preprocessing, the images were min–max normalized to be compatible with the network architecture (see Fig. 4). After detecting and cropping the coral images, that are scaled to 200 × 200 pixels preprocessed and labelled. See Fig. 5 for specific images for each coral species.

## Results

### Obtained by DL

High accuracy of 90% was obtained in a preliminary test by applying the DL method for classifying 400 images of four common coral species (see Supplementary Table 3).

Our method was demonstrated by applying training results from three sites to a fourth external site, reaching an overall 80.13% accuracy (2200 images of 11 coral classes were added for the fourth site). The results show accuracy of 80.13% for eleven coral species. The test data results show that the highest accuracy was observed for *Stylophora* (93.5%), *Lobophyllia* (92%) and *Montipora* (91.5%). Lower accuracies were obtained for *Platygyra* (89.5%), *Acropora* (81%), *Cyphastrea* (80%), *Porites* (74%), *Echinopora* (73%), *Pavona* (70.5%), *Goniastrea* (69.5%) and finally *Favia* (67%) (see Supplementary Table 4).

Using cross-validation results proves that the model can analyse new data from additional sites not used in the training (see Supplementary Tables 6 and 7).

### Obtained by traditional, non-DL methods

- There is no difference between the methods "Fiji ImageJ" and “Point estimated”, applied at each site (Cochran-Mantel–Haenszel test, X^2^ = 3.5084, p = 0.3197) (see supplementary statistical data Figure 6).
- There is a significant difference among live coral cover and the number of coral colonies for the four sites, by any method (see supplementary statistical data Figure 8).
- The difference in relative species’ coverage among the four sites was significant using both methods (see supplementary statistical data Figure 9).
- The species differed significantly in their coverage percentage. The coverage percentage among species differed statistically (One-way ANOVA, F(3,12) = 11.9, p = 0.000657) (see Supplementary Table 5).

## Discussion

The proposed computerized classification method can be configured to different characteristics of the dataset (e.g., size, number of classes, class types, etc.). We experimented with several CNN architectures, such as VGG-16 and ResNet-50, using also transfer learning. We applied ResNet-50 on a dataset of 5500 images to classify corals into 11 categories of coral species, by far the largest amount of images used in previous studies.

The classification of underwater coral images is challenging due to the large number of coral species, the great variance among images of the same coral, the lighting conditions, and the fact that several species tend to grow next to each other, leading to increasing overlapping among them. We demonstrated that the automatic classification obtained by a CNN of underwater coral images easily outperforms state-of-the-art, painstaking manual surveys (see Supplementary Table 3). ResNet-50 proved to perform the best, among the CNNs tested, due to its relative high speed, and level of accuracy (see Supplementary Table 4).

It is noteworthy that trained technicians or specialists can obviously identify many more than 11 species, as well as delete erroneous data, their training may take years. Furthermore, humans could never generate the vast amount produced by automated methods. Following the case presented by us, it is obvious that machine-based coral survey methods can be expanded to cover any coral species and non-species substrates^70^ (see review by Raphael, Dubinsky, Iluz and Netanyahu https://www.mdpi.com/1424-2818/12/1/29). We demonstrate the validity of existing automated surveying methods in an environment where such methods were not yet tested.

### Transfer learning

Due to the challenging problems facing coral reefs exposed to climate change and eutrophication, only a DL-based approach can provide the vast resources necessary to handle in real time, enormous high-resolution amounts monitoring data. This often requires the use of Transfer learning (TL), which is an ML technique that uses a model trained on one task to solve other tasks^71^ and additional new problems^72^. That technique works on condition that if the model features learned from the first task are generic enough to represent the features of the data seen during training.VGG-16 (very deep convolutional networks) is used in pre-trained models due to its high accuracy and advantages over ImageNet based classification.

Lumini et al.^73^ used DL based on CNN architectures for monitoring underwater ecosystems. In order to do so they used five well-known datasets (three of plankton and two of corals). They showed that their multiple models DenseNet succeeded the performance of the best single models. These authors used experimental data to examine the performance of both the single CNN and the ensemble of CNNs and showed that the best stand-alone model for most datasets was DenseNet.

The very deep convolutional network, used here VGG-16 is often used in pre-trained models due to its high accuracy and resolves the dataset classification problem inherent in ImageNet.

We used in this research a modified VGG-16 network, which essentially was pre-trained and tested successfully on the vast ImageNet dataset of millions of images from 1000 different categories, such as plants, animals, buildings, and humans.

The highly-accurate, 16-layer VGG-16 classification network is of size 528 Mbytes.

This modified architecture architecture resulted in an average 90% accuracy, i.e., much better than that obtained by running our images on the original network without any modifications, which would have given very poor results. In addition, the major benefit of using the VGG-16 network was in the remarkably short time required to train the dense layer, which is a fully-connected layer in which each unit or neuron is connected to each neuron in the next layer.

## Conclusions

### The innovations and accomplishments of this study

- This is the first study of its type done in the Gulf of Eilat.
- Will provide tools to follow the effect of climate change on the coral reefs of the Gulf of Eilat.
- Will allow the establishment of a baseline prior to the opening of the Red-Dead-Canal; real-time monitoring of its effects on the structure and biodiversity of the Gulf’s coral reefs.
- Foundation benchmark for the benefit of future studies done in the region.
- The refinement and development of the described DL method are applicable to reefs elsewhere.

The accomplishments of our work are by using “big data” in order to address the urgent ecological need of classifying corals, specifically those of the reefs in the Gulf of Eilat. We demonstrated the adaptation and application of Deep Learning Neuronal Networks for classifying corals in the Gulf of Eilat reefs. We applied DL to solve the problem of automated documentation of the structure of the coral reef at four sites in the Gulf of Eilat. Our study includes just corals and yet achieved accuracy similar to those that also included strikingly different classes, such as sea urchins, seaweeds, sand and bare ground.

### Future challenges in the application of DL to the study of reefs

- To develop the capability of DL for the study of time series in order to monitor and reveal temporal changes in the composition of reefs.
- To extract size/age distribution frequencies within single species populations.
- To document changes in live cover of corals in reefs.

## Supplementary information

Supplementary Information 1.

## Data Availability

The datasets generated during and/or analysed during the current study are available from the corresponding author on reasonable request.
