# Supplementary material for: Deep neural network recognition of shallow water corals in the Gulf of Eilat (Aqaba)
Source: Sci Rep. 2020 Jul 31;10:12959. doi: 10.1038/s41598-020-69201-w (PMC7395127; doi:10.1038/s41598-020-69201-w)
Supplement: Supplementary file 1 — Supplementary Information 1. [file 41598_2020_69201_MOESM1_ESM.docx]

**Deep neural network recognition of shallow water corals in the Gulf of Eilat (Aqaba)**

**Alina Raphael^1*^, Zvy Dubinsky^1^, David Iluz^1,3^, Jennifer I.C. Benichou^1^_,_ Nathan S. Netanyahu^2^**

^1^The Mina and Everard Goodman Faculty of Life Sciences, Bar-Ilan University,
Ramat-Gan 5290002, Israel

^2^Department of Computer Science, Bar-Ilan University, Ramat-Gan 5290002, Israel

^3^Department of Environmental Sciences and Agriculture, Beit Berl College, Beit Berl 4490500, Israel

**Corresponding author:** Alina Raphael

The Mina & Everard Goodman Faculty of Life Sciences

Bar-Ilan University

Ramat-Gan 5290002

Israel

Tel.: 972-3-972-3-5318283; Fax: 972-3-7384058; Email: creativeacs@gmail.com; [alina.raphael@live.biu.ac.il](mailto:alina.raphael@live.biu.ac.il)

**Supplementary Tables**

**Supplementary Table 1.** Gómez-Ríos at al. (2018) accuracies obtained on two datasets:

|  | **Shihavuddin's Method** | **Inception v3** | **ResNet-50** | **ResNet-152** | **DenseNet - 121** | **DenseNet - 161** |
| --- | --- | --- | --- | --- | --- | --- |
| Eilat | 95.79% | 95.25% | 97.85% | 97.85% | 91.03% | 93.81% |
| RSMAS | 92.74% | 96.03% | 97.67% | 97.95% | 89.73% | 91.10% |

**Supplementary Table 2.** Summary of methods for computer-assisted coral image classification.

Previous classiﬁcations using traditional machine learning methods.

| **First author (year)** | **Features** | **Classifiers** | **Number of classes** |
| --- | --- | --- | --- |
| **Mehta (2007)** | Color: RGB color space | Support vector machines SVM | 3  Classification of three types of corals of the genus corymbose *Acropora*: branching *Acropora*, *Acropora*, and tabulate *Acropora*. |
| **Marcos (2008)** | Color: Normalized chromaticity coordinates NCC histogram.  Texture: local binary pattern (LBP) | Linear discriminant analysis (LDA) | 5  Nonliving benthos are predominantly achromatic as compared to live benthos [living (L) and nonliving (N) components]. Traces of ground truth). |
| **Johnson – Roberson (2007)** | Color: RGB and HSV Texture: Gabor wavelets | Support vector machines SVM | 4  Discrimination of live coral from the seafloor. |
| **Purser (2009)** | Texture: Nonlinear Gabor filter | Artificial neural networks (ANN) | 3  Seabed cover by coral (*Lophelia pertusa*) or sponges (*Geodia baretti, Mycale lingua*). |
| **Stokes (2009)** | Color: Normalized RGB histogram  Texture: Discrete cosine transform (DCT) | Probability density whited mean distance (PDWMD) | 18  Library contains 18 different substrate/organism types, rather than the full complement of classified organisms from the manual processing dataset. |
| **Beijbom (2012)** | Color: L*A*B color space  Texture: Maximum response (MR) filter bank | Support vector machines (SVM) | 9  The coral genera *Acropora, Pavona, Montipora, Pocillopora, Porites* are marked by triangles, non-coral substrates, crustose coralline algae, turf algae, macroalgae and sand by circles. |
| **Stough (2012)** | Color: Quantile functions (QF)  Texture: Scale invariant feature transform (SIFT) | Linear support vector machines (SVM) | 2  Live *Acropora cervicornis* and all other content, including other live branching and nonbranching coral, dead *Acropora cervicornis*, algal cover, water, and hardground surfaces |

| **Shihavuddin (2013)** | Combination of:  Local binary pattern (CLBP)  Grey level co-occurrence matrix (GLCM)  Gabor filter response  Opponent angle and hue channel color histogram | Selection from:  K-nearest neighbor (KNN)  Neuronal network (NN)  Support vector machines (SVM)  Probability density whited mean distance (PDWMD) | 8  8 benthos types of classes on MLC dataset: sand, urchin, branches type I, brain coral, favid, branches type II, dead coral, and branches type III. Used six standard datasets (5-61) to compare the set of methods that are representative of the state-of-the-art in automated classification of seabed images. |
| --- | --- | --- | --- |

**Supplementary Table 3.** Summary of total accuracy.

| ***Acropora*** | ***Favia*** | ***Platygyra*** | ***Stylophora*** | **Total accuracy** | **Total false** |
| --- | --- | --- | --- | --- | --- |
| 93.33% | 93.33% | 80% | 93.33 | 90% | 10% |

**Supplementary Table 4.** Summary of deep learning test results of coral species.

| **Number** | **Coral Species** | **True** | **False** | **True (%)** |
| --- | --- | --- | --- | --- |
| 1 | *Acropora* | 162 | 17 | 81 |
| 2 | *Cyphastrea* | 160 | 93 | 80 |
| 3 | *Echinopora* | 146 | 27 | 73 |
| 4 | *Favia* | 134 | 81 | 67 |
| 5 | *Goniastrea* | 139 | 41 | 69.5 |
| 6 | *Lobophyllia* | 184 | 2 | 92 |
| 7 | *Montipora* | 183 | 68 | 91.5 |
| 8 | *Pavona* | 141 | 39 | 70.5 |
| 9 | *Platygyra* | 179 | 6 | 89.5 |
| 10 | *Porites* | 148 | 54 | 74 |
| 11 | *Stylophora* | 187 | 9 | 93.5 |

**Supplementary Table 5.** ANOVA test results for four coral species.

|  | **Df** | **Sum Sq.** | **Mean Sq.** | **F-value** | **Pr(>F)** |
| --- | --- | --- | --- | --- | --- |
| **Coral species** | 3 | 55.67 | 18.557 | 11.9 | 0.0006 |
| **Residuals** | 12 | 18.71 | 1.559 |  |  |

**Supplementary Table 6.** Cross validation accuracy results.

| **Cross Fold** | **Accuracy** |
| --- | --- |
| Cross_fold_0 | 81.54% |
| Cross_fold_1 | 82.27% |
| Cross_fold_2 | 82.81% |
| Cross_fold_3 | 81.54% |
| Cross_fold_4 | 81.90% |
| **Average Accuracy** | **82.01 %** |

**Supplementary Table 7.** Cross-Validation results.

|  | ***Acropora*** | ***Cyphastrea*** | ***Echinopora*** | ***Favia*** | ***Goniastrea*** | ***Lobophyllia*** | ***Montipora*** | ***Pavona*** | ***Platygyra*** | ***Porites*** | ***Stylophora*** |
| --- | --- | --- | --- | --- | --- | --- | --- | --- | --- | --- | --- |
| Fold-0 | 93 | 89 | 79 | 72 | 73 | 96 | 80 | 75 | 85 | 70 | 85 |
| Fold-1 | 91 | 86 | 69 | 74 | 80 | 99 | 80 | 78 | 87 | 77 | 84 |
| Fold-2 | 95 | 82 | 83 | 70 | 79 | 98 | 83 | 67 | 92 | 71 | 91 |
| Fold-3 | 90 | 84 | 73 | 76 | 71 | 93 | 81 | 79 | 88 | 71 | 91 |
| Fold-4 | 91 | 76 | 88 | 77 | 73 | 87 | 80 | 65 | 96 | 76 | 92 |
| **Average** | **92** | **83.4** | **78.4** | **73.8** | **75.2** | **94.6** | **80.8** | **72.8** | **89.6** | **73** | **88.6** |
| **Total Average** | | **82.01** | | | | | | | | | |

**References**

Beijbom O, Edmunds PJ, Kline DI, Mitchell BG, Kriegman D (2012) Automated annotation of coral reef survey images. IEEE Conference on Computer Vision and Pattern Recognition (CVPR)

Gomez-Rios A, Tabik S, Luengo J, Shihavuddin ASM, Krawczyk B, Herrera F (2018) Towards highly accurate coral texture images classification using deep convolutional neural networks and data augmentation. arXiv:1804.00516 [cs.CV]

Johnson-Roberson M, Kumar S, Williams S (2007) Segmentation and classification of coral for oceanographic surveys: A semi-supervised machine learning approach. OCEANS 2006 - Asia Pacific, May 16-19, 2007, Singapore

Marcos MSA, David L, Penaflor E, Ticzon V, Soriano M (2008) Automated benthic counting of living and non-living components in Ngedarrak Reef, Palau via subsurface underwater video. Environ Monit Assess 145:177-184

Mehta A, Ribeiro E, Gilner J, Van Woesik R (2007) Coral reef texture classification using support vector machines. VISAPP 2:302-310

Purser A, Bergmann M, Lundalv T, Ontrup J, Nattkemper TW (2009) Use of machine-learning algorithms for the automated detection of cold-water coral habitats: a pilot study. Mar Ecol Prog Ser 397:241-251

Shihavuddin ASM, Gracias N, Garcia R, Gleason AC, Gintert B (2013) Image-based coral reef classification and thematic mapping. Remote Sens 5:1809-1841

Stokes MD, Deane GB (2009) Automated processing of coral reef benthic images. Limnol Oceanogr Meth 7:157-168

Stough J, Greer L, Matt B (2012) Texture and color distribution-based classification for live coral detection Proceedings of the 12th International Coral Reef Symposium, pp 9-13

**Supplementary Statistical Data**

**Comparison between methods**

**Corals percentage coverage was measured in two different methods at four sites:**

General comparison between methods (sites=repeats)

Coral proportions were not significantly different between the two methods within different sites (Cochran-Mantel-Haenszel test, X^2^(3) = 3. 5084, p=0. 3197) (see Figure 6 and Figure 7).


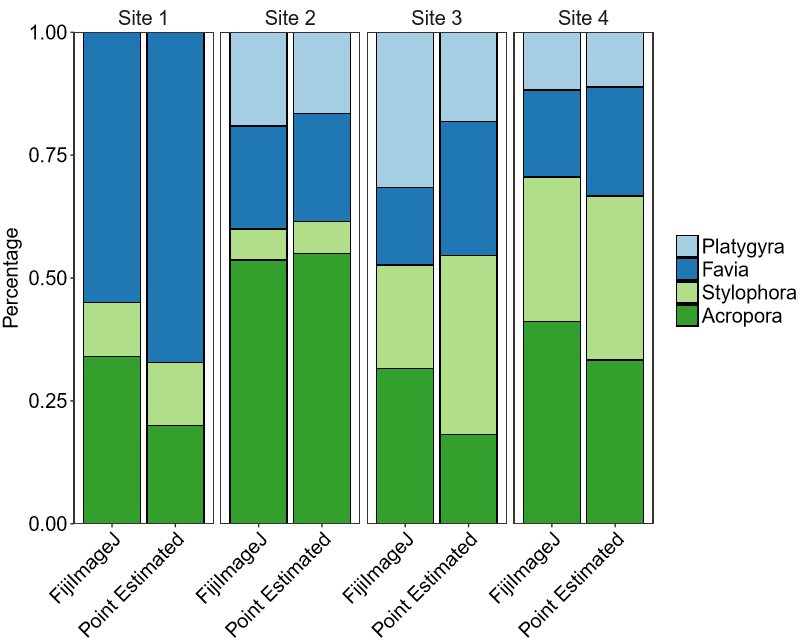


**[Figure 6](#toc):** The proportions of each coral in each method and on each site for four coral species (percent).


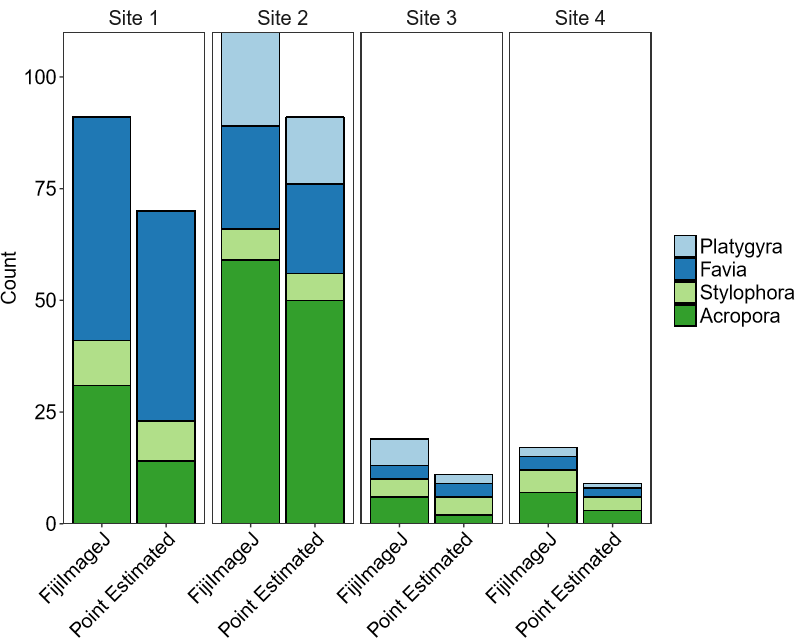


**Figure 7:** Counts of each coral in each method and on each site for four coral species.

**Comparison between sites**

General comparison between the sites taking into account the different methods (= repeats):

Proportions were significantly different between sites, within each method (Cochran-Mantel-Haenszel test, X^2^(9) = 110.67, p<0.0001) (see Figure 8).


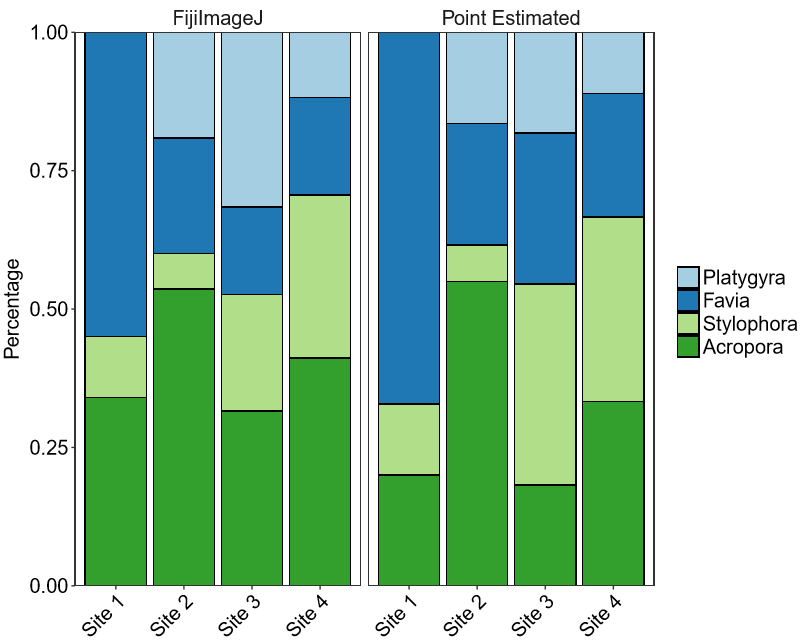

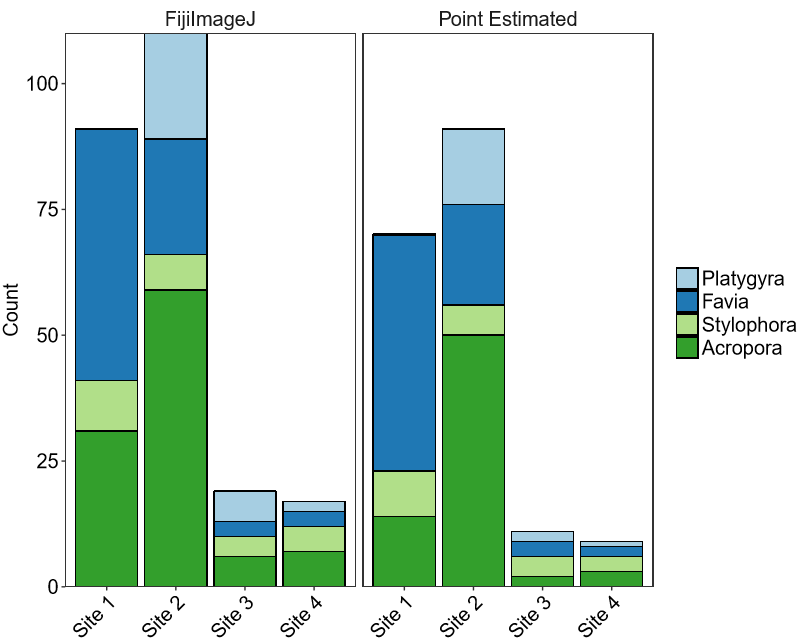


**a.**

**b.**

**Figure 8:** The proportions of each coral in each method and on each site for four coral species. (**a.** absolute numbers, **b.** percent).

**Comparison between each pair of sites for each method**

The Cochran-Mantel-Haenszel test was performed to compare proportions of corals that were measured with different methods across multiple sites, and proportions of corals in different sites within each method. Post hoc analysis was performed with pairwise Fisher test with FDR correction for multiple comparisons.

Coral proportions were not significantly different between the two methods within different sites (Cochran-Mantel-Haenszel test, Χ^2^(3)= 3.5084, p=0.3197). In contrast, proportions were significantly different between sites, within each method (Cochran-Mantel-Haenszel test, Χ^2^(9)= 110.67, p<0.0001). Specifically, proportions in site 1 was significantly different than proportions in sites 2 and 3 (p<0.0001) but there was no difference between sites 2 and 3 (p=0.0921) (see Table 6).

**Supplementary Table 6.** Comparison between each pair of sites for each method for four coral species.

| **Method** | **Sites** | **Adjusted P-value** |
| --- | --- | --- |
| FijiImageJ | 1-2 | < 0.001 |
| FijiImageJ | 1-3 | < 0.001 |
| FijiImageJ | 1-4 | 0.0254 |
| FijiImageJ | 2-3 | **0.0921** |
| FijiImageJ | 2-4 | **0.08** |
| FijiImageJ | 3-4 | **0.673** |
| Point Estimated | 1-2 | < 0.001 |
| Point Estimated | 1-3 | 0.0038 |
| Point Estimated | 1-4 | 0.0154 |
| Point Estimated | 2-3 | 0.0163 |
| Point Estimated | 2-4 | **0.1008** |
| Point Estimated | 3-4 | **0.9999** |


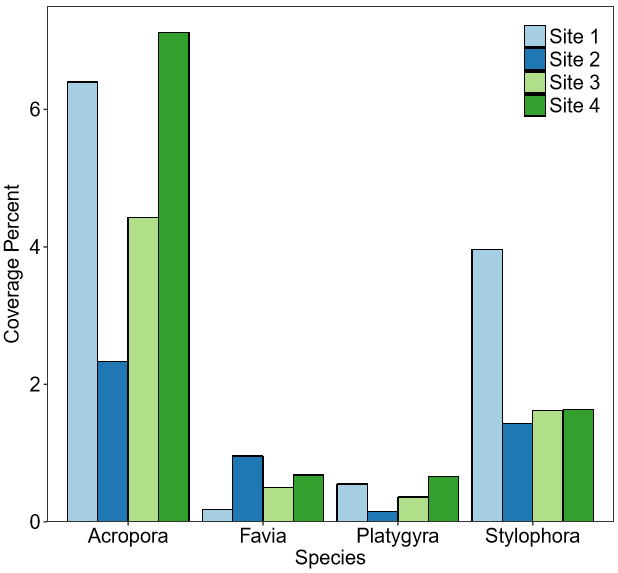
The highest coverage percentage in sites 1-4 was observed for *Acropora*. *Stylophora* has the highest coverage percentage in site one and the lowest in site 2. *Favia* has the highest coverage percentage in site 2 and the lowest in site 1; *Platygyra* has the highest coverage percentage in site 4 and the lowest in site 2 (see Figure 9).

**Figure 9:** Coverage percentage at each site for each coral species.

The coverage percentage of the common coral species in a descending order from the highest to the lowest was observed for *Acropora* , *Stylophora*, *Favia* and the lowest coverage percentage was found for *Platygyra* (see Figure10).


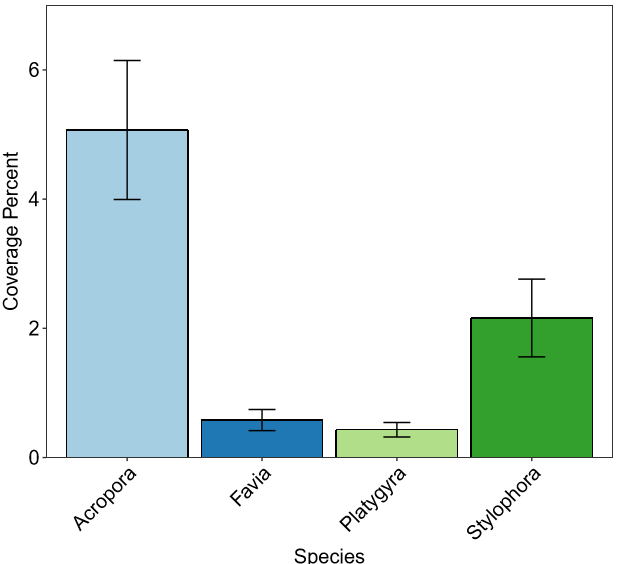


**Figure 10:** Coverage percentage of four coral species.

**Comparing the coverage percentages of different coral species**

5.1.4.1 One-way ANOVA test results

There was a statistically significant difference between the coverage percentage of the species

(One-way ANOVA, F(3,12) = 11.9, p = 0.000657) (see Table 7).

**Supplementary Table 7.** ANOVA test results for four coral species.

|  | **Df** | **Sum Sq.** | **Mean Sq.** | **F-value** | **Pr(>F)** |
| --- | --- | --- | --- | --- | --- |
| Coral species | 3 | 55.67 | 18.557 | 11.9 | 0.0006 |
| Residuals | 12 | 18.71 | 1.559 |  |  |

One-way ANOVA test shows significant differences.

**Tukey post hoc test**

A Tukey post hoc test showed that the coverage of *Acropora* was significantly higher than all other species (*Favia*- p=0.0013, *Platygyra*- p=0.001, *Stylophora*- p=0.028), but the coverage of

*Favia* was not significantly different than *Platygyra* and *Stylophora*.

There was no statistically significant difference between the coverage of *Platygyra* and

*Favia* (p = 0.998) and no statistically significant difference between the coverage of

*Stylophora-Favia* (p = 0.324) and no statistically significant difference between the coverage of

*Stylophora-Platygyra* (p=0.255) (see Table 8).

**Supplementary Table 8.** Multiple comparisons of means for four coral species.

| **Coral species** | **Mean Difference** | **95% Confidence Interval**  **of the Difference** | | **Adjusted P-value** |
| --- | --- | --- | --- | --- |
|  |  | **Lower** | **Upper** |  |
| *Favia-Acropora* | -4.49 | -7.111 | -1.868 | >0.001 |
| *Platygyra-Acropora* | -4.64 | -7.261 | -2.018 | 0.01 |
| *Stylophora-Acropora* | -2.91 | -5.531 | -0.288 | 0.028 |
| *Platygyra-Favia* | -0.15 | -2.771 | 2.471 | 0.998 |
| *Stylophora-Favia* | 1.58 | -1.041 | 4.201 | 0.324 |
| *Stylophora-Platygyra* | 1.73 | 0.891 | 4.351 | 0.255 |
